# Which theory is best? Explanatory models of the relationship between unemployment and health

**DOI:** 10.1186/1471-2458-9-235

**Published:** 2009-07-14

**Authors:** Urban Janlert, Anne Hammarström

**Affiliations:** 1 Department of Public Health and Clinical Medicine, Division of Public Health Sciences, Umeå University, Umeå, Sweden; 2 Department of Public Health and Clinical Medicine, Division of Family Medicine, Umeå University, Umeå, Sweden

## Abstract

**Background:**

A number of different models have been used in order to explain the links between unemployment and ill-health. The objective of this study was to test different proposed models in an empirical setting.

**Methods:**

A cohort of school-leavers consisting of more than 1000 persons was followed for 14 years up to the age of 30. They have repeatedly been asked questions that could be used to operationalise different proposed models as well as health outcomes. Seven different models explaining the health effect of unemployment were identified: an economic deprivation model, a lack of control model as well as a locus of control model, a stress model, a social support model, a work involvement model and a model of latent functions. Health outcomes used were somatic symptoms, depressive symptoms, self-perceived health and nervous problems. Statistical tests included bivariate correlations and logistic regression.

**Results:**

Most of the models correlated fairly well with unemployment measures. The capacity of the models to explain the connection between unemployment and ill-health varied, however. The model of latent functions was most successful, followed by the economic deprivation model. The social support and the control models were also fairly good. The work involvement scale and the stress model demonstrated the smallest explanatory power.

**Conclusion:**

Studies comparing different explanatory models in the field are rare. Few models apply a multidisciplinary approach. In view of the findings, it should be possible to develop multidisciplinary and better models to explain the links between unemployment and health in more detail.

## Background

Although seldom a prime objective for public health research, a number of models have been developed that explain the connection between unemployment and health [1].

Studies comparing these different models are lacking, however [2]. For this study we identified and tested seven different model concepts, namely: the *economic deprivation *model, the *lack of control *model as well as the *locus of control model*, the *stress *model, the *social support *model, the *work involvement model *and the model of *latent functions*.

Historically, three theoretical traditions can be traced with regard to unemployment research within the public health arena: i) the *biomedical *tradition, with its focus on physiological mechanisms to explain the correlation between mainly physical circumstances and biological phenomena (especially pathological phenomena); ii) the *sociological *tradition, with a focus on material circumstances creating both restrictions and possibilities for human development, and also an important formative environment for health and disease; and iii) the *psychological *tradition, focusing on the individual and his or her individual possibilities (perception, learning, motivation etc.)[3].

Proceeding from this, different combinations of traditions have been important in formulating new and more complex models. The stress model incorporates elements from both the biomedical and the psychological tradition; the economic deprivation models are mainly based on sociological theories but modelled on some psychological ideas; and the model of latent functions is a combination of sociological and psychological elements. From a public health point of view, all these theories need some reference to biological mechanisms.

The objective of this study was to test which model has the most powerful explanatory potential to clarify the connection between unemployment and health outcomes.

### The models

#### Economic deprivation models

This is the classical sociological model. Unemployed people will have less money, and less money will – directly or indirectly – worsen the prerequisites for good health. The model also suggests a potential solution to the problem: by giving the unemployed support for subsistence, the most deleterious effects of unemployment could be alleviated.

Studies utilising an economic deprivation model include most of the historical studies of the inter-war period [4]. Unemployed people lacked food, adequate housing and clothing. These studies followed a tradition from older investigations of pauperism and ill-health [5].

During the period of post-war unemployment, the economic situation was quite different, and unemployment benefits were the rule in most countries. Although the post-war unemployed were not as affluent as the employed, the economic conditions for the unemployed were substantially better than during the inter-war period. In spite of this, many studies still showed a persistent link between unemployment and ill-health [6]. Economic deprivation theory is still one of the dominant models in current studies [3,7,8].

#### Control models

These models encompass a wide variety of formulations, however they all state that the possibility to control (or feel that you can control) the environment is crucial to respond to a situation of unemployment.

The most widely used control concept in public health is the demand-control model, which was developed by Robert Karasek and combines job demands with decision latitude [9]. In the demand-control model unemployment can be regarded as a passive work situation, with low control and low demands in relation to working-life.

A specific aspect of control is the so-called locus of control, i.e. whether people feel that they are directed externally or internally [10]. An internal locus of control implies that the person can control the reinforcement him or herself, which means that the unemployed blame themselves for their lack of employment. Individuals with an external locus of control believe that reinforcement occurs by chance, and thus beyond one's own control. The unemployed therefore blame external forces for their situation. According to this hypothesis, those with an internal locus of control have a better chance of gaining employment as they feel that they themselves can control their situation [11].

#### Stress models

Although originally introduced by Walter Cannon, stress theory was popularised above all by Hans Selye. These models attempt to relate social stimuli to the health effects in the human being using physiological mechanisms as intermediating factors [12].

A theoretical model outlining the relationships between psychosocial stimuli and health outcome within the frame of stress theory was presented by French and Kahn [13]. Different versions of this model have been presented by many authors, including by Kagan and Levi [14].

In the stress models, psychosocial stimuli (e.g. employment termination) together with the psychobiological programme (including effects of earlier environmental and genetic factors) evoke the stress mechanism, which incidentally will result in precursors of disease. In more recent developments of the model, coping and social support play an important role in moderating the stress reaction [15].

Many unemployment studies have been carried out in this field. One of the classical plant closure studies, the so-called Michigan study from 1966, uses the stress concept explicitly [16].

An important part of the stress concept is the notion of "coping", i.e. how the individual handles the stress situation. In regards to unemployment research, only a few studies have focused on the effects of the coping process during unemployment [17].

#### Social support models

Theories of social support and social network are closely connected to the stress perspective. It is usual to differentiate between two different mechanisms for social support, the direct and the buffer effect. According to the direct effect model, lack of social network is supposed to have immediate consequences for health. The presence of human contact is looked upon as a fundamental need – when this is lacking it will result in unfavourable reactions. According to the buffer model, social support acts as a shield against different types of stress, e.g. unemployment.

House et al. conducted a study on effects of unemployment within this theoretical tradition [18]. Their analysis revealed only modest and selective effects of unemployment on social integration and support, however, on the other hand, social integration and support seemed most critical for promoting health and buffering the impact of unemployment. In a qualitative study by Thomas et al., it has even been suggested that unemployment has a positive effect on family relationships because of the increased time that the unemployed individual has to spend with their family [19].

#### Models of latent functions

The most renowned theory in this field is that of Marie Jahoda [20]. The idea behind these models is that work is supposed to contribute to a number of so-called latent functions. These latent functions include giving the day a time structure, providing opportunities for social contact with other people, contributing to status and personal identity for the individual, and providing an opportunity to strive towards collective purposes and shared experience. When these latent functions are lacking, ill-health may result.

Developments of this theory include the so-called vitamin model by Peter Warr [21]. This development has added other latent functions to the model and also modified some of the existing functions.

In a study of unemployed men in Brighton, United Kingdom, Ian Miles made explicit use of the concepts of Jahoda [22]. The study confirmed a strong connection between access to the five categories of experience and psychological well-being.

## Method

The basis for this analysis is a longitudinal cohort study, which has followed all pupils leaving mandatory school at age 16 in a medium-sized industrial town in Northern Sweden. The original participants were 1083 pupils all born in 1965, of which 574 were boys and 506 girls. They were first surveyed in 1981 at age 16. This study is based on a follow up survey in 1995 at 30 years of age. The attrition rate has been extremely low and, at this follow up, 96% of the original sample was still participating (547 men and 497 women). Divided into blue- and white-collar workers, 55% of the females and 50% of the males belonged to the blue-collar group. The study is described in detail elsewhere [23].

**Figure 1:**
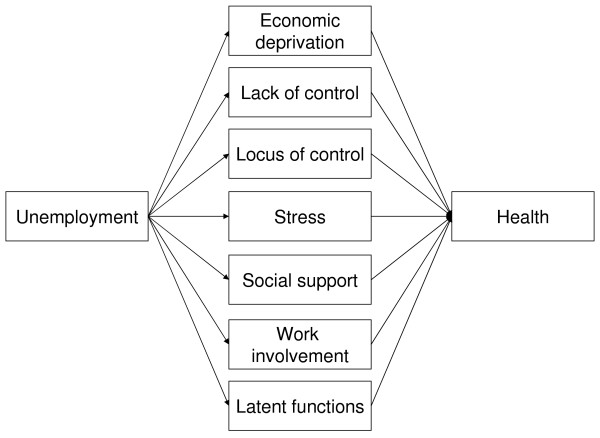
**Outline of the test design, where the middle boxes represent different explanatory models**.

Through the variables collected we were able to operationalise the different mechanisms that the various models are based on. As a first step we examine whether there are correlations between these models and unemployment, and as a second step what happens to the correlation between unemployment and health when the mechanisms are introduced as intervening variables (see Figure 1).

### Economic deprivation model

In order to characterise lack of money we used two different measures, namely: "cash margin", i.e. a question about whether the respondent could raise a certain amount of money within a week or not; and an index of economic stress.

The economic stress index consisted of a set of 11 questions and the respondents had to state whether they often, seldom or never had had to abstain from any of the following during the previous 12 months due to financial problems [24]:

• having a cooked meal

• buying clothes needed (for yourself or anybody else in the household)

• paying the rent or other bills

• going to cinema, concerts or theatres

• inviting friends to your home

• travelling to relatives or friends in other places

• buying presents which you would like to give away

• going on vacation

• subscribing to a paper

• partaking in leisure activities or hobbies

• going to restaurant or pub.

For every instance when the person responded "often", 1 was added to a total economic stress index, which thus could vary between 0 and 11.

### Lack of control

Lack of control was measured using questions from the control-demand questionnaire developed by Karasek and Theorell [9]. Instead of "work" we use the word "activity", which explicitly refers to the respondent's main activity, as paid work, home-work, studies, unemployment etc.

The following questions were used:

• Do you learn new things in your activity?

• Does your activity require skill?

• Does your activity require ingenuity?

• Does your activity require you to do the same things repeatedly?

• Have you freedom to decide how to do things in your activity?

• Have you freedom to decide what things should be done in your activity?

Each question was assigned a value from 1 (yes often) to 4 (no, practically never). The summarised measure (range 6 to 24) was used as an indicator of lack of control.

### Locus of control

Measurement of locus of control was based on two specific questions (with response alternatives "Agree fully", "Agree partly", "Don't agree"):

1) Unemployment is mainly due to individual factors (demand for work, education, resistance to migration or indolence)

2) Unemployment is mainly due to shortage of jobs in society.

External locus of control was defined as those who agreed fully with question 2, irrespective of their answer to question 1, and those who answered "Don't agree" to question 1 at the same time as answering "Agree partly" for question 2.

### Stress model

Stress was assessed in two different ways. From a set of questions about activity (work, home work, studies, unemployment etc.), those who indicated physical or psychological strain (heavy work load, high work intensity, psychological trying work, hard work, extensive work, work requiring high concentration, sleeping difficulties, strained and pressed situation) were selected to form two separate variables – two items indicating somatic strain and two indicating psychological strain. The range for both variables was 2–8.

### Social support

Social support was measured using an abbreviated form of the ISSI questionnaire, with two scales: a quantitative section, AVSI (availability of social interaction, four questions with range 4–24) and a qualitative section, AVAT (availability of attachment, six questions with range 6–22) [25].

### Work involvement

Work involvement was measured using the work involvement scale (WIS), which is a scale that has been developed to measure the inherent latent work functions according to Jahoda [26]. However, in the present study the scale was used separately and was not included in our measurement of the latent functions model. Examples of statements in the Work Involvement Scale are "It is very important for me to have a job", "Even if I won a large amount of money I would still like to work", or "Even if the dole was high, I would prefer working". There are six questions in the model with answers ranging from 1 (disagree completely) to 7 (agree completely). The variable thus has a range between 6 and 42.

### Latent functions according to Jahoda

We have identified five components in the so-called Jahoda model, namely:

• time structure

• social contacts

• collective purposes and shared experience

• status and personal identity

• regular activity.

**Time structure **was measured as the number of occurrences of structured and unstructured activities lasting at least four hours a week. The following activities were regarded as *structured*: work, studies, care of children, home work, maintenance of house and garden, handicraft, shopping and social activities. The following activities were regarded as *unstructured*: car travel (except going to work), hunting and fishing, physical training, pub and restaurant visits, cinema, theatre, reading, playing music, watching television, being together with friends and relatives. The structured variables had a range of 0–7, and the unstructured of 0–10.

**Social contacts **were measured using two separate variables: firstly with regard to being alone or together with others (range 0 to 1), and secondly being together in clubs and societies etc. (range 0 to 2).

**Collective purposes and shared experience **were measured using a set of seven questions about the workplace in relation to atmosphere, solidarity, comfort and well-being. The number of positive answers was summarised (range 0–7). Those who were unemployed were coded as 0 on this variable.

**Status and personal identity **were measured using four different questions. Positive answers to the following questions were summarised within the status variable (range 0–4):

• I am engaged with things that are important to have done

• I feel a strong affiliation with my relatives

• Most people appreciate what I do

• It is not very often I have problems in getting people to listen to what I have to say.

**Regular activity **was measured by three questions to which the respondent could give an answer between 1 (don't agree at all) and 7 (agree completely):

• I am fully occupied the whole time

• I always have to be very punctual

• Most of the day I have things to do at regular points in time.

The range of the variable was 3–21.

### Unemployment variable

The main independent variable used was total unemployment load (in weeks) during the observation period of the cohort, i.e. from age 16 to 30 (range 0–541 weeks). Unemployment was defined as a situation where a person had no job and was willing and able to take a job if offered. Persons studying or in labour market programmes were not considered to be unemployed.

In the correlation table (Table 1) the unemployment variable was used as a continuous variable. In the logistic regression tables (Tables 2, 3 and 4) the variable was dichotomised, so that unemployment for less than 1 year (52 weeks) was defined as not long-term unemployed, while those with more than 1 year of unemployment were defined as long-term unemployed.

**Table 1:** Bivariate correlations between indicators for the different models proposed in the literature and unemployment load (in weeks).

Variables	Women	Men
***Economic deprivation***	0.24**	0.29**
Cash margin	0.27**	0.30**
Economic stress	0.20**	0.27**
***Lack of control***	0.21**	***0.17***
Lack of control	0.21**	0.17**
***Locus of control***	0.19**	0.16**
Locus of control	0.19**	0.16**
***Stress model***	-0.16**	-***0.09***
Somatic strain	-0.14**	-0.05
Psychological strain	-0.17**	-0.13*
***Social support***	-***0.01***	***0.05***
Avsi	-0.23**	-0.09*
Avat	0.22**	0.14**
***Work involvement***	-0.16**	-0.08*
Work involvement scale	-0.16**	-0.18**
***Latent function model***	-0.15**	-0.08*
Structured work	-0.16**	-0.10*
Unstructured work	0.01	0.11*
Social contact 1	-0.14**	-0.10*
Social contact 2	0.02	0.11*
Collective purposes	-0.37**	-0.29**
Status	-0.21**	-0.26**
Regular activity	-0.21**	-0.05

Bold types indicate the correlation for the whole model. * = significant at 0.05 level, ** = significant at 0.01 level, two-sided tests.

**Table 2:** Bivariate correlations between the theoretical models and different health outcomes.

	Health outcome							
	Somatic symptoms		Depressive symptoms		Self-assessed health		Nervous problems	
Model	Women	Men	Women	Men	Women	Men	Women	Men
Economic depriv.	0.14**	0.16**	0.23**	0.29**	0.26**	0.17**	0.12**	0.19**
Lack of control	0.20**	0.14**	0.18**	0.17**	0.17**	0.15**	0.20**	0.12**
Locus of control	0.02	-0.01	0.05	0.04	0.10*	0.05	0.01	0.03
Stress	0.05	0.15**	-0.02	0.01	0.06	0.07	0.13**	0.01
Social support	-0.01	0.03	0.10*	0.03	-0.04	0.12**	0.07	0.08
Work involvem.	-0.10*	-0.01	-0.04	-0.05	-0.06	-0.07	-0.06	-0.06
Latent function	-0.12**	-0.03	-0.11*	-0.17**	-0.13**	-0.13**	-0.06	-0.14**

* = significant at 0.05 level, ** = significant at 0.01 level, two-sided tests.

**Table 3:** OR for somatic symptoms among long-term unemployed (> 1 year) compared to not long-term unemployed for different explanatory models.

	Women		Men	
Independent variable set	OR	95% CI	OR	95% CI
Only unemployment	2.64	1.57–4.44	2.18	1.41–3.35
Unemployment + WIS	2.94	1.71–5.02	2.19	1.40–3.41
Unemployment + Stress model	2.84	1.66–4.85	2.25	1.42–3.56
Unemployment + Locus of control	2.67	1.58–4.51	2.23	1.44–3.45
Unemployment + Social support	2.34	1.35–4.05	2.15	1.37–3.38
Unemployment + Lack of control	2.19	1.26–3.80	2.20	1.41–3.43
Unemployment + Economic deprivation	2.25	1.32–3.85	1.88	1.20–2.93
Unemployment + latent functions	1.70	0.92–3.15	2.16	1.29–3.61

**Table 4:** OR for depression among long-term unemployed (> 1 year) compared to not long-term unemployed for different explanatory models.

	Women		Men	
Independent variable set	OR	95% CI	OR	95% CI
Only unemployment	2.07	1.15–3.73	2.21	1.15–2.24
Unemployment + WIS	2.03	1.11–3.72	2.02	1.02–4.02
Unemployment + Locus of control	2.01	1.13–3.65	2.13	1.11–4.13
Unemployment + Stress model	1.69	0.88–3.28	2.22	1.11–4.43
Unemployment + Economic deprivation	1.65	0.89–3.04	1.30	0.63–2.65
Unemployment + Social support	1.49	0.77–2.88	1.64	0.80–3.36
Unemployment + Lack of control	1.51	0.78–2.93	2.07	1.04–4.15
Unemployment + Latent functions	0.97	0.44–2.14	0.82	0.34–2.02

### Outcome variables

In order to test the different mechanisms, four different health outcomes at age 30 were used. *Somatic symptoms *are identified on the basis of affirmative answers to a list of 34 different somatic symptoms. Respondents were instructed to tick the box if they had had any of the listed symptoms during the previous 12 months. If the number of symptoms was 6 or more (for women 8 or more), the "symptoms" variable was regarded as positive, and otherwise negative. *Depressive symptoms *were identified according to positive answers ("rather often" or "always") to the question of how often they had been depressed or in a low mood during the previous 12 months. Poor *self-perceived health *was measured by the answer "bad" or "between bad and good" for the question "How do you assess your health?". *Nervous problems *were measured as a "yes" answer to the question "Have you had nervous problems during the last 12 months?". Somatic and depressive symptoms both correlated significantly with unemployment and were used in the analyses for both men and women. Self-perceived health only correlated significantly with unemployment for women and nervous problems only for men, and were thus only analysed for women and men respectively.

### Statistics

The idea of a model is that it should guide research in that area, summarise the findings and make correlations found in a study causally plausible [4]. The better the model, the more the correlation between unemployment and ill-health can be captured. From a technical point of view, a good model should also explain the correlation by including the critical variables that mediate the connection.

Logistic regression has been used to test the different models. If the odds ratio (OR) came closer to 1 it was interpreted that the model in question contained variables that could explain partly the connection between unemployment and the outcome. If the OR was 1, there was no remaining variation to be explained.

### Ethical clearance

The study was approved by the Research Ethics Committees at Uppsala and Umeå Universities.

## Results

Table 1 shows the results of the bivariate correlations between the different model indicators and the unemployment measure (number of weeks in unemployment) during the period 1981–95. Results are shown separately for men and women. Of 32 tested correlations, 27 were found to be significant, most of them at the 0.01 confidence level.

Table 2 shows the corresponding correlations between the different models and the four outcomes – somatic symptoms, depressive symptoms, self-assessed health and nervous problems.

Table 3 presents the results of a number of logistic regressions with somatic symptoms as the dependent variable, and the different sets of mechanisms as the independent together with the unemployment variable. Each row represents a separate analysis.

In the first test only the unemployment variable was entered, giving an odds ratio that does not include 1. When the work involvement scale was used as the independent variable together with unemployment, the odds ratio was even higher than with only unemployment, showing that the work involvement variable had another explanatory direction than the supposed one.

The only mechanism that could reduce the odds ratio with regard to somatic symptoms to an insignificant level was the latent functions model by Jahoda, and that was only for women. When using all the variables in Table 1 as independent variables, the odds ratio was higher than when using the latent functions variables.

Table 4 presents the same analysis as Table 3, but here with depression as the outcome variable. The procedure is repeated in Table 5, but here with self-rated health (women) and nervous problems (men).

**Table 5:** OR for bad self-rated general health for women and nervous problems for men among long-term unemployed (> 1 year) compared to not long-term unemployed for different explanatory models.

	Women (self-rated health)		Men (nervous problems)	
Independent variable set	OR	95% CI	OR	95% CI
Only unemployment	1.86	1.23–2.81	1.98	1.29–3.04
Unemployment + Stress model	1.95	1.26–3.01	2.18	1.39–3.42
Unemployment + Work involvement scale	1.81	1.19–2.75	1.91	1.23–2.98
Unemployment + Locus of control	1.78	1.17–2.70	2.03	1.32–3.13
Unemployment + Lack of control	1.78	1.15–2.75	2.03	1.36–3.15
Unemployment + Social support	1.59	1.03–2.46	1.87	1.19–2.92
Unemployment + Economic deprivation	1.55	1.01–2.39	1.72	1.10–2.60
Unemployment + Latent functions	1.15	0.70–1.89	1.49	0.90–2.49

Comparing the three different outcomes, we see that the reduction of OR is lowest with the work involvement scale, the stress model and the locus of control. At an intermediate OR level we find social support, lack of control and economic deprivation models. In all three analyses we find that the model with latent functions is the most effective in explaining the correlations.

Ranking the outcome (with the best outcome being an OR as close to 1 as possible) using the six separate tests (one for each sex presented in tables 2, 3 and 4) we find that the theory of latent functions has the lowest summarised rank score (9), closely followed by the economic deprivation model (13). Following this comes social support (18), control model (22), locus of control (32), work involvement scale (33) and finally the stress model (39). If we only consider the tests where the model was successful in producing a non-significant OR, the latent structure model did so in five out of six tests, followed by the economic deprivation model, the control model and the social support model (all with two non-significant outcomes) and the stress model (one non-significant). The locus of control model and work involvement scale did not give a non-significant result in any test.

When analysing the five constructs of the latent function model separately we find that time structure and regular activity were the components (for both women and men) that had the highest OR for the different outcomes, while collective purposes and shared experience (women) and status and personal identity (men) showed the weakest connection.

## Discussion

### Synopsis of findings

The present study shows the capacity of various models to explain the connection between unemployment and health outcome. With regard to outcome, depression seems to be the best outcome variable that the different models are able to explain. For women only the work involvement scale and the locus of control model were unsuccessful, and for men the locus of control also and the stress model.

Considering the latent function model specifically, it seems that time related components (time structure, regular activities) had the strongest explanatory power, while more socially oriented variables (collective purposes, status, personal identity as well as shared experiences) were weaker.

### Contribution to existing literature

Few previous studies have tried to test different explanatory models on the same data set. Our analyses show that the explanatory power differs between the models, but also between different outcomes.

### Methodological issues

The aim of the present study was to test the different models per se, which is why we do not include a number of alternative hypotheses, e.g. reversed causation. No adjustment was made for social class, as our measure of unemployment is highly correlated with class.

The different models include different numbers of variables, and it could be assumed that the more variables, the better the fit. This seems to apply regarding the latent functions model, in which six variables were included compared to only two in the social support model. The economic deprivation model consisted of only two items, however, and was fairly successful.

The construction of the different explanatory variables in the models sometimes violates strict statistical criteria regarding the level of the scale. However, sensitivity tests, where calculations were made with dichotomous scales, did not yield different results in principle regarding the statistical significant figures. We therefore believe that our results will not be affected by these circumstances.

A limitation of this study is that the different models have been constructed using available data in an existing data set, rather than one that has been set up specifically for the purpose of this study. The empirical data used were therefore not ideal from the perspective of the different models, and it is also probable that existing data covered certain models better than others. However, even if the variables are more or less representative of the models, the ranking of the explanatory power seems to be approximately the same, irrespective of outcome, which suggests some robustness. We also believe that a study such as this should use data collected primarily for other purposes, and that it should be supplemented with other studies based on other data sources.

### Future research

The overall impression is that model development is limited to specific disciplines and that multidisciplinary theory development is rare. There is a need for development of broader and more complex theories in order to better understand the determinants of ill-health among the unemployed. If, on the other hand, prediction is the goal, a more parsimonious model would be preferable. In which case, economic deprivation, lack of control and social support could be models that are good enough to reveal something about the risk of health effects, and perhaps also to provide policy guidance.

Multidisciplinarity is not a characteristic of any of the tested models. As we see it, no model encompasses more than two different disciplinary approaches. A hypothesis that could be raised is whether a more multidisciplinary model is better suited to explaining the connection between unemployment and health. This could be tested in a new study with a focus on models incorporating different discipline-specific components.

## Conclusion

Of the five models tested, most correlated fairly well with unemployment measures. The capacity of the models to explain the connection between unemployment and ill-health varied, however. The model of latent functions was the most successful model, followed by the economic deprivation model. The social support, as well as the control models, were also fairly good. The work involvement scale and the stress model demonstrated the smallest explanatory power. In view of these findings it should be possible to develop more specific and better models to explain the links between unemployment and ill-health.

## Abbreviations

AVAT: availability of attachment; AVSI: availability of social interaction; CI: confidence interval; ISSI: Interview Schedule for Social Interaction; OR: odds ratio; WIS: work involvement scale.

## Competing interests

The authors declare that they have no competing interests.

## Authors' contributions

AH and UJ designed the study and AH had main responsibility for data collection with support from UJ. UJ carried out all statistical analyses with support from AH. UJ wrote a draft of the manuscript, which was commented upon by AH. Both authors have read and approved the final manuscript.

## Pre-publication history

The pre-publication history for this paper can be accessed here:


